# Ubiquitin C-Terminal Hydrolase L1 in Tumorigenesis

**DOI:** 10.1155/2012/123706

**Published:** 2012-07-01

**Authors:** Jennifer Hurst-Kennedy, Lih-Shen Chin, Lian Li

**Affiliations:** Department of Pharmacology and Center for Neurodegenerative Disease, Emory University School of Medicine, Atlanta, GA 30322, USA

## Abstract

Ubiquitin carboxyl-terminal hydrolase L1 (UCH-L1, aka PGP9.5) is an abundant, neuronal deubiquitinating enzyme that has also been suggested to possess E3 ubiquitin-protein ligase activity and/or stabilize ubiquitin monomers *in vivo*. Recent evidence implicates dysregulation of UCH-L1 in the pathogenesis and progression of human cancers. Although typically only expressed in neurons, high levels of UCH-L1 have been found in many nonneuronal tumors, including breast, colorectal, and pancreatic carcinomas. UCH-L1 has also been implicated in the regulation of metastasis and cell growth during the progression of nonsmall cell lung carcinoma, colorectal cancer, and lymphoma. Together these studies suggest UCH-L1 has a potent oncogenic role and drives tumor development. Conversely, others have observed promoter methylation-mediated silencing of UCH-L1 in certain tumor subtypes, suggesting a potential tumor suppressor role for UCH-L1. In this paper, we provide an overview of the evidence supporting the involvement of UCH-L1 in tumor development and discuss the potential mechanisms of action of UCH-L1 in oncogenesis.

## 1. Introduction

Ubiquitin carboxyl-terminal hydrolase L1 (UCH-L1, aka PGP9.5) is an abundant neuronal protein consisting of 223 amino acids [1]. The best understood function of UCH-L1 is its deubiquitinating enzyme (DUB) activity that catalyzes hydrolysis of C-terminal esters and amides of ubiquitin (Ub) to generate monomeric Ub [2, 3]. In addition to its DUB activity, UCH-L1 has also been suggested to possess a putative, dimerization-dependent E3 ubiquitin-protein ligase activity and/or have a role in stabilizing Ub monomers *in vivo *[4, 5]. As a DUB, UCH-L1 facilitates Ub recycling and, therefore, can regulate the cellular pool of available Ub [6], giving UCH-L1 the capacity to modulate many ubiquitin-dependent cellular processes. Although its exact physiological function remains unclear, a growing body of evidence implicates UCH-L1 in the progression of human malignancies. Currently, the specific role of UCH-L1 in cancer pathogenesis is not known. UCH-L1 has been reported to be upregulated in several tumor tissues and cancer cell lines [7–13] and has been suggested to function as an oncogene in the progression of many cancers including lymphoma [11], colorectal cancer [14], and nonsmall cell lung carcinoma [8]. Conversely, studies have been put forth designating UCH-L1 as a tumor suppressor in the pathogenesis of nasopharyngeal [15] and breast [16] cancer. Despite the controversy regarding the exact function of UCH-L1 in oncogenesis, these studies suggest that UCH-L1 is an important regulator of tumor formation and maturation. Here, we review the current knowledge of the function and mechanisms of actions of UCH-L1 in tumorigenesis. 

## 2. Functions of UCH-L1 in the UPS

The ubiquitin-proteasome system (UPS) is a major intracellular proteolytic pathway that facilitates the degradation of normal cellular proteins as well as the clearance of misfolded and damaged proteins [17]. In the UPS, protein substrates are tagged with polymers of a 76-amino-acid polypeptide, ubiquitin (Ub), followed by recognition and degradation by the 26S proteasome. This process is facilitated by the sequential actions of at least three classes of enzymes: ubiquitin-activating enzymes (E1), ubiquitin-conjugating enzymes (E2), and ubiquitin-protein ligases (E3). First, an E1 activates Ub at the expense of ATP. Next, activated Ub is transferred to an E2 enzyme. Finally, an E3 specifically recognizes its protein substrate, which can be in its normal conformation or misfolded, and catalyzes the transfers of activated Ub from an E2 to the substrate. Successive addition of Ub to a lysine residue of a previously conjugated Ub results in the formation of a polyubiquitin chain. K48-linked polyubiquitin chains serve as a recognition signaling for proteasomal degradation. Once ubiquitinated substrates are transferred to the proteasome, DUBs remove the Ub chain, allowing for free Ub monomers to be recycled. Monoubiquitination and noncanonical polyubiquitination (e.g., K63 ubiquitin linkages) of proteins have been implicated in nonproteasomal cellular processes, including endocytosis, trafficking, cell signaling, DNA damage repair, and modifications of histones [17, 18].

UCH-L1 was first identified as a member of the ubiquitin carboxyl-terminal hydrolase (UCH) family of DUBs with cysteine protease activity in the late 1980s [1]. UCH-L1 is an abundant neuronal protein, comprising approximately 2% of total brain protein [1, 19]. Although low levels of UCH-L1 protein have been reported to be present in kidneys, breast epithelium, and reproductive tissues [20, 21], UCH-L1 is absent in most other tissues [1, 19, 22, 23]. UCH-L1 appears to play an important role in neurons, as mice lacking functional UCH-L1 have been reported to exhibit neuronal dysfunction and neurodegeneration [24, 25]. At the subcellular level, UCH-L1 is primarily found in the cytoplasm [26], but recent reports indicate that a subpopulation of UCH-L1 can be transiently localized to the nucleus [13, 27]. Biochemical studies revealed that UCH-L1 hydrolyzes Ub at its C-terminal glycine residue to generate monomeric Ub *in vitro* [1] and that this activity is dependent upon the catalytic residues C90 and H161 [2]. Analysis of UCH-L1 crystal structure indicates that these catalytic residues are not accessible to large polymers of Ub and suggest that UCH-L1 preferentially binds monomeric Ub and small adducts of Ub [28]. It is possible that substrate binding and/or the presence of cofactors may induce a conformational change, allowing UCH-L1 to process larger Ub chains. However, this has not yet been demonstrated *in vitro* or *in vivo*. Thus, UCH-L1 is best understood to function as a cysteine protease capable of hydrolyzing small Ub moieties. 

**Figure 1:**
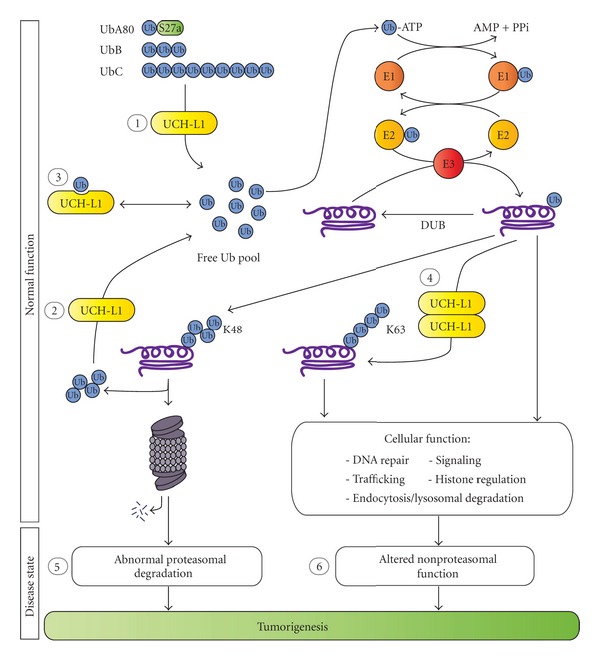
Molecular functions of ubiquitin c-terminal hydrolase L1. (1) UCH-L1 can hydrolyze ubiquitin pro-proteins to generate monomeric ubiquitin (Ub) [29]. (2) UCH-L1 may also facilitate Ub recycling by processing Ub chains. (3) UCH-L1 has been reported to stabilize monomeric Ub by binding to Ub and preventing its degradation by the lysosome [4]. Collectively, these functions (1, 2, and 3) give UCH-L1 control over the availability of free Ub and, therefore, the potential to influence many ubiquitination-dependent cellular processes, including proteasomal degradation, DNA damage repair, trafficking, cell signaling, endocytosis, and lysosomal degradation. (4) Dimerized UCH-L1 may possess ATP-independent E3 ligase activity that facilitates K63-linked polyubiquitination [5], although it is currently unclear whether this putative E3 ligase activity directly regulates ubiquitination of protein substrates *in vivo*. (5) Altered expression of UCH-L1 may cause changes to the free Ub pool, resulting in abnormal K48-linked polyubiquitination and proteasomal degradation. (6) Changes in the free Ub pool may also affect mono- and K63-linked polyubiquitination, leading to altered nonproteasomal functions and tumorigenesis.

Although the exact function of UCH-L1 is not fully understood, several studies suggest that UCH-L1 regulates the cellular pool of free Ub (Figure 1). First, UCH-L1 has been reported to cleave the ubiquitin gene products UbB and UbC and the ribosomal ubiquitin fusion protein UbA80 to generate monomeric Ub [29], leading to an increase in the level of free Ub (Figure 1). UCH-L1 may also elevate free Ub levels by facilitating recycling of Ub (Figure 1). Next, it has also been suggested that UCH-L1 plays a role in stabilizing Ub monomers by binding to monomeric Ub and preventing its lysosomal degradation (Figure 1) [4]. Association of UCH-L1 with monomeric Ub occurs independently of the catalytic C90 residue, indicating that mono-Ub binding is not dependent upon UCH-L1 hydrolase activity [4]. The role of UCH-L1 in the regulation of the free Ub pool is also supported by the observation that levels of monomeric Ub are decreased in gracile axonal dystrophy (*gad*) mice, which lack functional UCH-L1 [4] In contrast to other DUBs, *in vitro* studies indicate that UCH-L1 does not directly catalyze the deubiquitination of ubiquitinated protein substrates [29]. Moreover, no *in vivo* UCH-L1 substrates have been identified thus far. Collectively, current evidence suggests that UCH-L1 functions to increase the cellular pool of free Ub by hydrolyzing small Ub chains and stabilizing monomeric Ub rather than by directly acting on polyubiquitinated substrates.

UCH-L1 has been reported to possess putative, dimerization-dependent E3 ligase activity in addition to its hydrolase function (Figure 1) [5]. *In vitro* studies show that dimeric UCH-L1 promotes K63-linked polyubiquitination of *α*-synculein [5]. Unlike other E3 ligases, UCH-L1 E3 ligase activity was observed in the absence of ATP [5], which differs from the mechanism of conventional ubiquitination [17, 18]. It is currently not known whether UCH-L1 exhibits E3 ligase activity *in vivo*. Further investigation into UCH-L1 enzymatic function is needed to understand its role in health and disease.

## 3. UCH-L1 as a Positive Regulator of Tumorigenesis 

Although UCH-L1 is almost exclusively expressed in neurons [1, 19], proteomic screens have revealed that UCH-L1 is present in many nonneuronal human tumors (Table 1) including adenocarcinoma [35], pancreatic ductal carcinoma [36], and squamous cell carcinoma [31]. Similarly, microarray profiling analyses show UCH-L1 mRNA is upregulated in several breast cancer tumor types [37] and medullary thyroid carcinoma tumors [38]. UCH-L1 mRNA has also been shown to be elevated in gall bladder and colorectal tumor tissues as a result of hypomethylation of the UCH-L1 promoter [39, 40]. High levels of UCH-L1 protein have also been observed in many human tumor-derived cell lines (Table 1) such as those cultured from lung [8], prostate [41, 42], and bladder tumors [43] as well as B-cell lymphomas [44] and osteosarcomas [45]. The presence of UCH-L1 in nonneuronal tumor tissues and cancer cell lines suggests that increased levels of UCH-L1 may promote oncogenic transformation and, therefore, point to a possible role for UCH-L1 as an oncogene in cancer pathogenesis. 

The potential oncogenic function of UCH-L1 is supported by a number of clinical studies demonstrating that UCH-L1 expression level in tumors is inversely correlated with patient survivability [14, 36, 37]. High levels of UCH-L1 mRNA in breast tumors have been reported to be associated with poor prognosis in patients [37]. Likewise, elevated UCH-L1 mRNA in colorectal tumors is associated with higher incidence of tumor recurrence and shorter survival time [14]. Moreover, UCH-L1 expression in pancreatic ductal tumors is correlated with decreased patient survival [36]. Together, these data suggest that UCH-L1 is involved in tumor maturation.

To determine whether upregulation of UCH-L1 is a result of oncogenic transformation or itself a driving force of tumorigenesis, the direct involvement of UCH-L1 in cancer pathogenesis has been investigated. *In vitro* tumorigenesis studies show that UCH-L1 stimulates oncogenic transformation and invasion in nonsmall cell lung carcinoma [8] and colorectal cancer [14] cells, suggesting that UCH-L1 may function as an oncogene in these cancers. Furthermore, Hussain et al. have demonstrated that transgenic mice constitutively expressing UCH-L1 under the control of a CAGGS promoter form sporadic tumors in all tissues [11]. Of these tumors, lymphomas are the most prevalent [11]. Further investigation revealed that shRNA-mediated knock down of UCH-L1 in immortalized B cells decreased cell growth and viability, suggesting UCH-L1 promotes the development of lymphomas by inhibiting cell death and by stimulating proliferation [11]. Collectively, these data suggest UCH-L1 is a potent oncogene with the capacity to promote tumorigenesis in many different cell types. 

Recently, it has been suggested that UCH-L1 promotes cancer cell motility and invasion, which may contribute to its oncogenic role. Overexpression of UCH-L1 in HCT8 colorectal cancer cells has been reported to enhance cell migration [9]. Additionally, Kim et al. have shown that siRNA-mediated knock down of UCH-L1 reduces H157 lung carcinoma cancer cells migration *in vitro* [8]. They further demonstrated that depletion of UCH-L1 attenuates lung metastasis *in vivo* in a murine xenograft model [8]. UCH-L1 stimulates prostate cancer cell migration and invasion as well by promoting epithelial-to-mesenchymal transition (EMT) [41]. UCH-L1 level also appears to be correlated with cancer cell metastatic capacity. While UCH-L1 is found in many lung carcinoma cell lines, it is further upregulated in high metastatic lines [8]. Likewise, low metastatic LNCaP and RWPE1 prostate cancer cells do not express UCH-L1, while high metastatic DU145 prostate cancer cells abundantly express UCH-L1 [41]. These studies suggest that UCH-L1 promotes cancer cell metastasis. Further studies are needed to determine how UCH-L1 regulates cell motility and invasion.

Despite growing evidence implicating UCH-L1 as a positive regulator of tumor growth and development, the mechanism by which UCH-L1 conveys oncogenesis is not fully understood. Many of the investigations into the role of UCH-L1 in cancer have focused on upregulation of UCH-L1 in tumor tissues and cancer cells. However, little is known about changes in UCH-L1 enzymatic activity during tumorigenesis. Although one group has observed a decrease UCH-L1 hydrolase activity in cervical carcinoma tissues and an increase in hydrolase activity in transformed keratinocytes [12], the role of UCH-L1 enzymatic function(s) in cancer is largely unknown. Furthermore, no evidence of genetic amplification of UCH-L1 or oncogenic mutations in UCH-L1 have been reported to date, although a Parkinson's disease-linked mutation has been identified [51]. Elucidation of UCH-L1 enzymatic activity in tumorigenesis and investigation into oncogenic genetic alterations of UCH-L1 may provide insights into the role of this enzyme in cancer pathogenesis.

## 4. UCH-L1 as a Potential Tumor Suppressor

In contrast to the body of literature identifying UCH-L1 as an oncogene, several reports have been put forth suggesting UCH-L1 acts as a tumor suppressor during the pathogenesis of certain cancers [10, 16, 46, 50]. Contrary to previous reports proposing UCH-L1 enhances the progression of prostate cancer [41, 42], two recent studies from Ummanni et al. suggest that UCH-L1 attenuates prostate tumor growth and maturation [46, 50]. UCH-L1 may possibly act as a tumor suppressor in breast cancer pathogenesis as well [16, 52]. In contrast to previous studies demonstrating that UCH-L1 is upregulated in breast tumors [21, 37], UCH-L1 mRNA expression was reported to be decreased in several breast carcinoma cell lines [16]. Moreover, ectopic expression of UCH-L1 in breast cancer cells caused a decrease in anchorage-independent cell growth and an increase apoptosis, suggesting UCH-L1 may act as a negative regulator of breast tumorigenesis [16, 52]. UCH-L1 has also been implicated in the suppression of nasopharyngeal carcinoma as UCH-L1 mRNA expression is decreased in many nasopharyngeal tumors [10]. Lastly, UCH-L1 promoter methylation is elevated in malignant prostate tumors [46], primary breast tumors [16], and nasopharyngeal carcinomas [10]. Similarly, several breast cancer [16] and gastric cancer cell lines [34] exhibit enhanced methylation of UCH-L1 promoter sequences, resulting in decreased UCH-L1 transcription (Table 1). Taken together, these observations suggest that UCH-L1 may function as a tumor suppressor, particularly in prostate [46, 50], breast [16, 52], and nasopharyngeal [10] carcinomas.

There are a number of possible reasons for the discrepancies in the observed oncogenic and tumor suppressor functions of UCH-L1 in tumorigenesis. First, studies suggesting UCH-L1 attenuates prostate cancer progression [46, 50] focused on the behavior of low metastatic prostate cancer cells, while those implicating UCH-L1 as a positive regulator of prostate tumorigenesis [41, 42] investigated more mature prostate tumors and cell lines. Whether UCH-L1 elicits different effects as prostate tumors become more malignant remains to be investigated. Next, many studies have suggested UCH-L1 functions as a tumor suppressor based on observed decreases in UCH-L1 mRNA in tumor tissues and transformed cells [10, 16, 34, 47, 48]. However, it is not known whether differences in UCH-L1 transcription in these tumors and cells result in changes in UCH-L1 protein level and/or UCH-L1 enzymatic activity. Finally, UCH-L1 is absent or expressed at very low levels in all nonneuronal tissues [1, 19, 22, 23], raising an important question regarding the reported tumor suppressor role for UCH-L1: how can a reduction in UCH-L1 mRNA in tissues that normally express little to no UCH-L1 protein convey oncogenic transformation? To address this question, the normal expression pattern and/or physiological role of UCH-L1 in nonneuronal tissues need to be clarified.

## 5. Potential Mechanisms of Actions of UCH-L1 in Tumorigenesis

**Figure 2:**
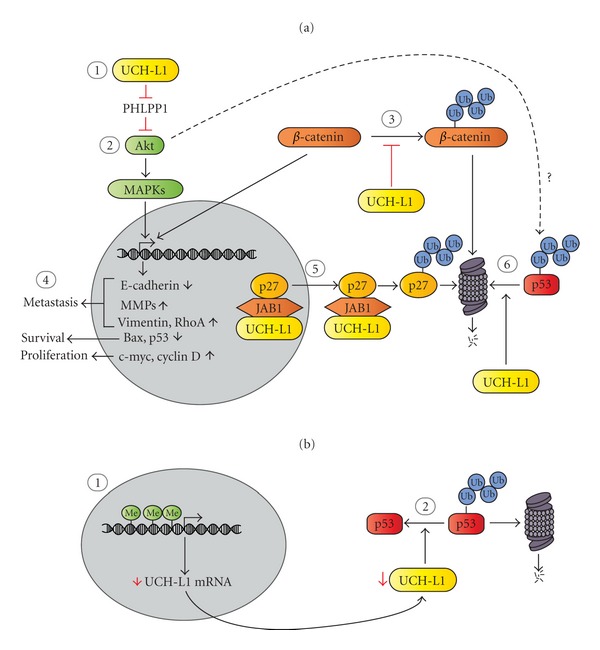
The potential roles of UCH-L1 in tumorigenesis. (a) UCH-L1 as a possible oncogene that promotes metastasis and cell growth. (1) UCH-L1 is up-regulated in several tumor tissues and cancer cell lines [7–13]. (2) Elevated UCH-L1 may stimulate Akt through inhibition of the phosphatase PHLLP1 [11], leading to increased MAPK signaling [8]. (3) UCH-L1 has been reported to decrease polyubiquitination and proteasomal degradation of *β*-catenin, resulting in enhanced *β*-catenin-mediated transcription [30]. (4) Increased *β*-catenin and Akt signaling could potentially cause changes in gene transcription that promote metastasis and proliferation and inhibit apoptosis, resulting in enhanced oncogenicity [31–33]. (5) UCH-L1 binds to JAB1 and promotes the nuclear export and subsequent proteasomal degradation of the cell cycle inhibitor p27 [13]. (6) Upregulation of UCH-L1 has been reported to promote proteasomal degradation of p53 [11], which may be a consequence of activation of Akt signaling. Reduction of p27 and p53 levels by UCH-L1 may attenuate cell cycle arrest, allowing for uncontrolled cell growth. (b) UCH-L1 as a putative tumor suppressor in certain cancer subtypes. (1) Reduction of UCH-L1 transcription via promoter methylation-silencing has been observed in certain cancer cells and tumor tissues (e.g., nasopharyngeal carcinomas [10] and gastric cancer cells [34]). (2) In these cancer types, it has been proposed that UCH-L1 promotes deubiquitination of p53 and inhibits its proteasomal degradation [10, 16]. Reduced UCH-L1 transcription due to promoter methylation may thus lead to increased degradation of p53, resulting in a reduction of p53-mediated transcription of tumor suppressing genes and enhanced tumorigenesis (see text for more details).

Currently, the precise mechanism(s) of UCH-L1-mediated tumorigenesis are not fully understood. Previous studies have identified several cancer-related signaling processes that are regulated by UCH-L1, which may contribute to its role in oncogenesis. In particular, UCH-L1 has been implicated in the regulation of cell cycle progression, cell survival, cell motility, and invasion (Figure 2).

### 5.1. UCH-L1 Enzymatic Activity and Oncogenesis

Disruption of UPS function has been implicated in cancer pathogenesis and progression [53] and many cancer-related cellular processes are controlled by ubiquitination, including cell division, growth factor signaling, DNA damage repair, and apoptosis [54–57]. As previously stated, UCH-L1 hydrolyzes small Ub molecules to generate free Ub and also stabilizes monomeric Ub [4, 29] (Figure 1). Through these functions, UCH-L1 can increase the free pool of Ub and, therefore, indirectly affect many ubiquitination-dependent cellular activities. In a pathogenic state, UCH-L1 dysfunction has the potential to alter the cellular levels of monomeric Ub, possibly causing global changes in protein ubiquitination. Therefore, aberrant UCH-L1 signaling may indirectly alter both the poly- and monoubiquitination of oncogenes and tumor suppressors, possibility leading to abnormal protein degradation and/or altered protein function and subsequent tumorigenesis (Figure 1). 

UCH-L1 has also been reported to promote K63-linked polyubiquitination of *α*-synuclein through its putative, dimerization-dependent E3 ubiquitin ligase activity [5]. K63-linked polyubiquitination has been implicated in cancer-related cellular processes such as DNA damage repair and cell survival signaling [58, 59]. Although UCH-L1 E3 activity has not been observed *in vivo* and substrates other than *α*-synuclein have not been identified, alterations in UCH-L1 function may disrupt K63-linked polyubiquitination, possibly altering nonproteasomal cellular processes to promote tumorigenesis (Figure 1). Further investigation is needed to clarify the potential E3 function of UCH-L1 as well as the normal physiological and oncogenic role of this enzymatic function. 

### 5.2. A Possible Function for UCH-L1 in Cell Cycle Regulation

UCH-L1 has been shown to stimulate proliferation in transformed lymphocytes and cervical carcinoma cells [11, 27], while it promotes G1/S arrest in breast cancer cells [16]. Together, these studies imply that UCH-L1 contributes to cancer pathogenesis by regulating cell division, although the exact control that UCH-L1 confers on the cell cycle remains unclear. UCH-L1 has been shown to modulate the levels of several cell cycle regulators in cancer cells including cyclin D [31] and p53 [10]. Coimmunoprecipitation experiments conducted by Caballero et al. have shown that UCH-L1 also interacts with JAB1 (Jun-activation domain-binding protein 1). Binding of UCH-L1 to JAB1 promotes the nuclear export and subsequent proteasomal degradation of the cyclin dependent kinase inhibitor p27 [13], resulting in increased cell proliferation (Figure 2(a)). These observations suggest UCH-L1 controls cell cycle progression by modulating the availability of cell cycle regulatory proteins, possibly by altering their ubiquitination status. Recent evidence implicates UCH-L1 in the regulation of cell cycle progression via direct interactions with microtubules. Bheda et al. have demonstrated that UCH-L1 is tightly associated with microtubules during cell division in several transformed cell lines, and that siRNA-mediated knockdown of UCH-L1 reduces microtubule assembly and disassembly [27]. Interestingly, both 25 kDa and 50 kDa UCH-L1 species were associated with purified microtubules [27], suggesting UCH-L1 may act as a dimer to regulate microtubule dynamics. Taken together, these observations suggest that UCH-L1 regulates cell cycle progression by altering levels of cell cycle regulatory proteins and by controlling microtubule dynamics. However, further studies are needed to determine the specific manner by which UCH-L1 controls cell division. In particular, whether or not UCH-L1 controls ubiquitination of cell cycle regulators should be examined.

### 5.3. UCH-L1 and Cell Survival Signaling

Overactivation of the serine-threonine kinase Akt is a common hallmark of cancer pathogenesis [60]. Phosphorylation of Akt leads to activation of several signaling cascades that together promote cell survival by stimulating proliferation and inhibiting apoptosis. Pharmacological inhibitors of Akt kinase activity attenuate UCH-L1-mediated ECM invasion in nonsmall cell lung carcinoma cells [8]. Additionally, overexpression of UCH-L1 in these cells increases phosphorylation of the downstream Akt targets p38 and ERK1/2, suggesting that UCH-L1 promotes cell survival through Akt-dependent activation of MAPK signaling [8]. Lastly, overexpression of UCH-L1 in immortalized B cells also has been shown to increase Akt phosphorylation during lymphoma progression [11]. Together, these data suggest UCH-L1 elicits at least some of its cellular effects through Akt-dependent signaling and that stimulation of Akt by UCH-L1 is a potential mechanism of UCH-L1-mediated oncogenesis (Figure 2(a)). UCH-L1 promotes Akt signaling, in part, by reducing the level of the tumor suppressor PHLPP1 [11], a phosphatase that reverses Akt phosphorylation rendering Akt inactive. However, the mechanism by which UCH-L1 suppresses PHLPP1 merits further investigation as UCH-L1 does not alter PHLPP1 transcription or promote proteasomal degradation of PHLPP1. Furthermore, whether or not UCH-L1 modulates upstream activators of Akt remains to be determined. Nevertheless, stimulation of Akt by UCH-L1 and the subsequent promotion of prosurvival signaling may contribute to the function of UCH-L1 in oncogenesis.

A number of studies suggest that UCH-L1 exerts its actions through regulation of the tumor suppressor p53. However, the specific manner in which UCH-L1 modulates p53 level and function remains controversial. UCH-L1 has been shown to promote the proteasomal degradation of p53 in HeLa cells [11], and microarray analyses conducted by Bheda et al. show that depletion of UCH-L1 in 293T cells increases the levels of many p53 target genes [32], suggesting UCH-L1 suppresses p53 signaling. On the other hand, overexpression of UCH-L1 was reported to increase p53 levels in MDA-MB-231 breast carcinoma cells [16] and HONE1 nasopharyngeal carcinoma cells [10]. Similarly, Li et al. have shown that over-expression of UCH-L1 in LNCaP prostate cancer cells reduces polyubiquitination of p53, leading to inhibition of degradation of p53 by the proteasome [50]. They also observed an increase in polyubiquitination and degradation of mdm2 in response to UCH-L1 over-expression, suggesting UCH-L1 suppresses mdm2 to stabilize p53 levels [50].

Further studies are needed to determine specifically how UCH-L1 modulates p53. Discrepancies in observed regulation of p53 by UCH-L1 may be attributed to differences in p53 status. Studies implicating UCH-L1 as a negative regulator of p53 [11, 32] were conducted in cells with wild-type p53 [61, 62]. On the contrary, UCH-L1 elevates p53 levels in MDA-MB-231 and HONE1 cells, which express DNA binding domain mutant p53 with little to no transcriptional activity [63–65] and LNCaP cells, which have also been reported to express DNA binding domain mutant p53 [66], although this is controversial [67].This suggests that UCH-L1 may regulate wild-type and DNA binding domain mutant p53 differently. P53 is frequently mutated in human cancers [63] and variation in p53 status may offer another possible explanation for why UCH-L1 has been reported to function as an oncogene and a tumor suppressor in different cancer cell lines and tumor types. It is possible that in cells with wild type p53, UCH-L1 promotes degradation p53, resulting in reduced p53 signaling and inhibition of cell death (Figure 2(a)). On the other hand, in other cell types with weakened p53 transcriptional activity, UCH-L1 may regulate nontranscriptional functions of p53 [68, 69] to promote apoptosis and attenuation of tumor growth (Figure 2(b)). It is also possible that UCH-L1 indirectly elicits control over p53 by modulating negative regulators of p53, such as mdm2, as suggested by Li et al. [10]. As p53 level and function are regulated, in part, by ubiquitination [58, 70, 71], investigation into modulation of p53 ubiquitination by UCH-L1 may offer additional insights into the role of UCH-L1 in tumorigenesis. However, while exploring the relationship between UCH-L1 and p53 ubiquitination, it is important to keep in mind that, despite hypotheses to the contrary [10, 16], it is unlikely that UCH-L1 directly deubiquitinates or ubiquitinates p53 based on what is known about UCH-L1 structure and function [28, 29]. Additionally, it might be possible that activation of Akt signaling by UCH-L1 [8, 11] might also contribute to its control over p53, as Akt is an established negative regulator of p53 activity [60, 72].

### 5.4. The Potential Role of UCH-L1 in Metastasis

UCH-L1 has been suggested to promote metastasis in colorectal, lung, and prostate cancer cells [8, 9, 41]. Cancer cell metastasis is often attributed to hyperactivation of *β*-catenin, a transcription factor that when over-activated promotes cell migration and invasion [73]. UCH-L1 overexpression has been shown decrease polyubiquitination and proteasomal degradation of *β*-catenin in HEK 293 cells, leading to stabilization of TCF: *β*-catenin complexes and increased *β*-catenin-mediated transcription of prosurvival genes such as c-myc, c-jun, and survivin [30]. Although, it is unlikely UCH-L1 directly deubiquitinates *β*-catenin [28, 29], these observations suggest UCH-L1 may convey its oncogenic function through Wnt signaling pathways. UCH-L1 itself has been identified as a target of *β*-catenin-mediated transcription, suggesting there is a positive feedback loop between *β*-catenin and UCH-L1 that enhances metastasis [30]. One consequence of *β*-catenin signaling is promotion of epithelial-to-mesenchymal transition (EMT) [33]. Recently, it was shown that UCH-L1 enhances prostate cancer cell metastasis by increasing the expression of pro-EMT genes such as vimentin and matrix metalloproteinases (MMPs) and reducing transcription of the EMT suppressor E-cadherin [41]. Together, these data imply that UCH-L1 promotes cancer cell metastasis via *β*-catenin-induced EMT (Figure 2(a)). Therefore, therapeutic targeting of Wnt and EMT signaling may prove to be an effective treatment for tumors that express high levels of UCH-L1.

## 6. Conclusions

In summary, emerging evidence suggests that UCH-L1 is a potent oncogene that promotes tumor growth and development during the progression of many forms of cancer. However, the exact role of UCH-L1 in oncogenesis remains controversial, as UCH-L1 has been suggested to function as a tumor suppressor in certain tumor types. The observed involvement of UCH-L1 in the regulation of cell cycle progression, cell survival, and metastasis may explain its oncogenic role. However, further studies are needed to clarify the exact mechanisms of action of UCH-L1 in tumorigenesis. Continued investigation into the function of UCH-L1 in cancer may tell us whether or not UCH-L1 can be used as a diagnostic marker. UCH-L1 is upregulated in many cancer tissues and, therefore, high levels of UCH-L1, particularly in nonneuronal tissues, may serve as an early detection biomarker for tumors. Furthermore, UCH-L1 itself could be a potential therapeutic target, which may have benefits for the treatment of cancer. Elucidation of the role of UCH-L1 in cancer may lead to a better understanding of the molecular pathogenesis of tumors as well as potentially facilitate the development of novel cancer therapeutics and diagnostics tools.

## Figures and Tables

**Table 1:** Aberrant expression of UCH-L1 in tumor tissues and cancer cells.

Elevated UCH-L1	Down-Regulated UCH-L1
Malignant Tumors	

Squamous cell carcinoma [31]	Prostate tumors [46]
Medullary thyroid carcinoma tumors [38]	Primary breast cancer tumors [16]
Osteosarcoma [45]	Primary nasopharyngeal carcinoma [10]
Adenocarcinoma [35]	Colorectal carcinoma [47]
Metastatic colorectal cancer tumors [9]	Melanoma [48]
Breast cancer tumors [37]	Diffuse-type gastric cancer [34]
Pancreatic ductal carcinoma tumors [36]	
Parathyroid carcinoma [49]	

Transformed Cells	

SaOS-2 osteosarcoma cells [45]	LNCaP prostate cancer cells [50]
BLZ-211 and BLS-211 bladder cancer cells [43]	
BL30, X-50/7, KR4, Raji, KR4 B-cell lymphoma cells [44]	
HCT8 colorectal cancer cells [9]	
DU154 prostate cancer cells [41, 42]	
H157, W138, H358 lung carcinoma cells [8]	

## Abbreviations

UCH-L1: Ubiquitin C-terminal hydrolase L1
Ub: Ubiquitin
UPS: Ubiquitin-proteasome system
DUB: Deubiquitinating enzyme
E1: E1 Ubiquitin-activating enzyme
E2: E2 Ubiquitin-conjugating enzyme
E3: E3 Ubiquitin-protein ligase
Akt: Protein Kinase B
MAPKs: Mitogen-activated protein kinases
ERK1/2: Extracellular signal-related kinases 1 and 2
PHLPP1: PH domain leucine-rich repeat protein phosphatase 1
JAB1: Jun-activation domain-binding protein 1
EMT: Epithelial-to-mesenchymal transition
MMPs: Matrix metalloproteinases.

## References

[1] Wilkinson KD, Lee K, Deshpande S, Duerksen-Hughes P, Boss JM, Pohl J (1989). The neuron-specific protein PGP 9.5 is a ubiquitin carboxyl-terminal hydrolase. *Science*.

[2] Larsen CN, Price JS, Wilkinson KD (1996). Substrate binding and catalysis by ubiquitin C-terminal hydrolases: identification of two active site residues. *Biochemistry*.

[3] Case A, Stein RL (2006). Mechanistic studies of ubiquitin C-terminal hydrolase L1. *Biochemistry*.

[4] Osaka H, Wang YL, Takada K (2003). Ubiquitin carboxy-terminal hydrolase L1 binds to and stabilizes monoubiquitin in neuron. *Human Molecular Genetics*.

[5] Liu Y, Fallon L, Lashuel HA, Liu Z, Lansbury PT (2002). The UCH-L1 gene encodes two opposing enzymatic activities that affect *α*-synuclein degradation and Parkinson’s disease susceptibility. *Cell*.

[6] Walters BJ, Campbell SL, Chen PC (2008). Differential effects of Usp14 and Uch-L1 on the ubiquitin proteasome system and synaptic activity. *Molecular and Cellular Neuroscience*.

[7] Liu Y, Lashuel HA, Choi S (2003). Discovery of inhibitors that elucidate the role of UCH-L1 activity in the H1299 lung cancer cell line. *Chemistry and Biology*.

[8] Kim HJ, Kim YM, Lim S (2009). Ubiquitin C-terminal hydrolase-L1 is a key regulator of tumor cell invasion and metastasis. *Oncogene*.

[9] Ma Y, Zhao M, Zhong J (2010). Proteomic profiling of proteins associated with lymph node metastasis in colorectal cancer. *Journal of Cellular Biochemistry*.

[10] Li L, Tao Q, Jin H (2010). The tumor suppressor UCHL1 forms a complex with p53/MDM2/ARF to promote p53 signaling and is frequently silenced in nasopharyngeal carcinoma. *Clinical Cancer Research*.

[11] Hussain S, Foreman O, Perkins SL (2010). The de-ubiquitinase UCH-L1 is an oncogene that drives the development of lymphoma *in vivo* by deregulating PHLPP1 and Akt signaling. *Leukemia*.

[12] Rolén U, Kobzeva V, Gasparjan N (2006). Activity profiling of deubiquitinating enzymes in cervical carcinoma biopsies and cell lines. *Molecular Carcinogenesis*.

[13] Caballero OL, Resto V, Patturajan M (2002). Interaction and colocalization of PGP9.5 with JAB1 and p27Kip1. *Oncogene*.

[14] Akishima-Fukasawa Y, Ino Y, Nakanishi Y (2010). Significance of PGP9.5 expression in cancer-associated fibroblasts for prognosis of colorectal carcinoma. *American Journal of Clinical Pathology*.

[15] Li L, Tao Q, Jin H (2010). The tumor suppressor UCHL1 forms a complex with p53/MDM2/ARF to promote p53 signaling and is frequently silenced in nasopharyngeal carcinoma. *Clinical Cancer Research*.

[16] Xiang T, Li L, Yin X (2012). The ubiquitin peptidase UCHL1 induces G0/G1 cell cycle arrest and apoptosis through stabilizing p53 and is frequently silenced in breast cancer. *PLoS One*.

[17] Li L, Chin L-S (2007). Impairment of the ubiquitin-proteasome system: a commnon pathogenic mechansim in neurodegenerative disorders. *The Ubiquitin Proteasome System*.

[18] Pickart CM, Fushman D (2004). Polyubiquitin chains: polymeric protein signals. *Current Opinion in Chemical Biology*.

[19] Wilson POG, Barber PC, Hamid QA (1988). The immunolocalization of protein gene product 9.5 using rabbit polyclonal and mouse monoclonal antibodies. *British Journal of Experimental Pathology*.

[20] Bradbury JM, Thompson RJ (1985). Immunoassay of the neuronal and neuroendocrine marker PGP 9.5 in human tissues. *Journal of Neurochemistry*.

[21] Schumacher U, Mitchell BS, Kaiserling E (1994). The neuronal marker protein gene product 9.5 (PGP 9.5) is phenotypically expressed in human breast epithelium, in milk, and in benign and malignant breast tumors. *DNA and Cell Biology*.

[22] Kajimoto Y, Hashimoto T, Shirai Y, Nishino N, Kuno T, Tanaka C (1992). cDNA cloning and tissue distribution of a rat ubiquitin carboxyl-terminal hydrolase PGP9.5. *Journal of Biochemistry*.

[23] Day INM, Thompson RJ (2010). UCHL1 (PGP 9.5): neuronal biomarker and ubiquitin system protein. *Progress in Neurobiology*.

[24] Sakurai M, Sekiguchi M, Zushida K (2008). Reduction in memory in passive avoidance learning, exploratory behaviour and synaptic plasticity in mice with a spontaneous deletion in the ubiquitin C-terminal hydrolase L1 gene. *European Journal of Neuroscience*.

[25] Saigoh K, Wang YL, Suh JG (1999). Intragenic deletion in the gene encoding ubiquitin carboxy-terminal hydrolase in gad mice. *Nature Genetics*.

[26] Lowe J, McDermott H, Landon M, Mayer RJ, Wilkinson KD (1990). Ubiquitin carboxyl-terminal hydrolase (PGP 9.5) is selectively present in ubiquitinated inclusion bodies characteristic of human neurodegenerative diseases. *Journal of Pathology*.

[27] Bheda A, Gullapalli A, Caplow M, Pagano JS, Shackelford J (2010). Ubiquitin editing enzyme UCH L1 and microtubule dynamics: implication in mitosis. *Cell Cycle*.

[28] Messick TE, Russell NS, Iwata AJ (2008). Structural basis for ubiquitin recognition by the Otu1 ovarian tumor domain protein. *Journal of Biological Chemistry*.

[29] Larsen CN, Krantz BA, Wilkinson KD (1998). Substrate specificity of deubiquitinating enzymes: ubiquitin C-terminal hydrolases. *Biochemistry*.

[30] Bheda A, Yue W, Gullapalli A (2009). Positive reciprocal regulation of ubiquitin C-terminal hydrolase L1 and *β*-catenin/TCF signaling. *PLoS ONE*.

[31] Mastoraki A, Ioannidis E, Apostolaki A, Patsouris E, Aroni K (2009). PGP 9.5 and cyclin D1 coexpression in cutaneous squamous cell carcinomas. *International Journal of Surgical Pathology*.

[32] Bheda A, Shackelford J, Pagano JS (2009). Expression and functional studies of ubiquitin C-terminal hydrolase L1 regulated genes. *PLoS ONE*.

[33] Rozenberg GI, Monahan KB, Torrice C, Bear JE, Sharpless NE (2010). Metastasis in an orthotopic murine model of melanoma is independent of RAS/RAF mutation. *Melanoma Research*.

[34] Yamashita K, Park HL, Kim MS (2006). PGP9.5 methylation in diffuse-type gastric cancer. *Cancer Research*.

[35] Chen G, Gharib TG, Huang CC (2002). Proteomic analysis of lung adenocarcinoma: identification of a highly expressed set of proteins in tumors. *Clinical Cancer Research*.

[36] Tezel E, Hibi K, Nagasaka T, Nakao A (2000). PGP9.5 as a prognostic factor in pancreatic cancer. *Clinical Cancer Research*.

[37] Miyoshi Y, Nakayama S, Torikoshi Y (2006). High expression of ubiquitin caboxy-terminal hydrolase-L1 and -L3 mRNA predicts early recurrence in patients with invasive breast cancer. *Cancer Science*.

[38] Takano T, Miyauchi A, Matsuzuka F (2004). PGP9.5 mRNA could contribute to the molecular-based diagnosis of medullary thyroid carcinoma. *European Journal of Cancer*.

[39] Lee YM, Lee JY, Kim MJ (2006). Hypomethylation of the protein gene product 9.5 promoter region in gallbladder cancer and its relationship with clinicopathological features. *Cancer Science*.

[40] Mizukami H, Shirahata A, Goto T (2008). PGP9.5 methylation as a marker for metastatic colorectal cancer. *Anticancer Research A*.

[41] Jang MJ, Baek SH, Kim JH (2011). UCH-L1 promotes cancer metastasis in prostate cancer cells through EMT induction. *Cancer Letters*.

[42] Leiblich A, Cross SS, Catto JWF, Pesce G, Hamdy FC, Rehman I (2007). Human prostate cancer cells express neuroendocrine cell markers PGP 9.5 and chromogranin A. *Prostate*.

[43] Yang Y-C, Li X, Chen W (2006). Characterization of genes associated with different phenotypes of human bladder cancer cells. *Acta Biochimica et Biophysica Sinica*.

[44] Bheda A, Yue W, Gullapalli A, Shackelford J, Pagano JS (2011). PU.1-dependent regulation of UCH L1 expression in B-lymphoma cells. *Leukemia and Lymphoma*.

[45] Liu X, Zeng B, Ma J, Wan C (2009). Comparative proteomic analysis of osteosarcoma cell and human primary cultured osteoblastic cell. *Cancer Investigation*.

[46] Ummanni R, Mundt F, Pospisil H (2011). Identification of clinically relevant protein targets in prostate cancer with 2D-DIGE coupled mass spectrometry and systems biology network platform. *PLoS ONE*.

[47] Fukutomi S, Seki N, Koda K, Miyazaki M (2007). Identification of methylation-silenced genes in colorectal cancer cell lines: genomic screening using oligonucleotide arrays. *Scandinavian Journal of Gastroenterology*.

[48] Bonazzi VF, Nancarrow DJ, Stark MS (2011). Cross-platform array screening identifies COL1A2, THBS1, TNFRSF10D and UCHL1 as genes frequently silenced by methylation in melanoma. *PLoS ONE*.

[49] Adam MA, Untch BR, Olson JA (2010). Parathyroid carcinoma: current understanding and new insights into gene expression and intraoperative parathyroid hormone kinetics. *Oncologist*.

[50] Ummanni R, Jost E, Braig M (2011). Ubiquitin carboxyl-terminal hydrolase 1 (UCHL1) is a potential tumour suppressor in prostate cancer and is frequently silenced by promoter methylation. *Molecular Cancer*.

[51] Nishikawa K, Li H, Kawamura R (2003). Alterations of structure and hydrolase activity of parkinsonism-associated human ubiquitin carboxyl-terminal hydrolase L1 variants. *Biochemical and Biophysical Research Communications*.

[52] Wang WJ, Li QQ, Xu JD (2008). Over-expression of Ubiquitin carboxy terminal hydrolase-L1 induces apoptosis in breast cancer cells. *International Journal of Oncology*.

[53] Newton K, Vucic D (2007). Ubiquitin ligases in cancer: ushers for degradation. *Cancer Investigation*.

[54] Li M, Brooks CL, Wu-Baer F, Chen D, Baer R, Gu W (2003). Mono- versus polyubiquitination: differential control of p53 Fate by Mdm2. *Science*.

[55] Ea CK, Deng L, Xia ZP, Pineda G, Chen ZJ (2006). Activation of IKK by TNF*α* requires site-specific ubiquitination of RIP1 and polyubiquitin binding by NEMO. *Molecular Cell*.

[56] Moynahan ME, Chiu JW, Koller BH, Jasint M (1999). Brca1 controls homology-directed DNA repair. *Molecular Cell*.

[57] Mamillapalli R, Gavrilova N, Mihaylova VT (2001). PTEN regulates the ubiquitin-dependent degradation of the CDK inhibitor p27KIP1 through the ubiquitin E3 ligase SCFSKP2. *Current Biology*.

[58] Wen R, Li J, Xu X, Cui Z, Xiao W (2012). Zebrafish Mms2 promotes K63-linked polyubiquitination and is involved in p53-mediated DNA-damage response. *DNA Repair*.

[59] Harhaj EW, Dixit VM (2012). Regulation of NF-kappaB by deubiquitinases. *Immunological Reviews*.

[60] Carnero A (2010). The PKB/AKT pathway in cancer. *Current Pharmaceutical Design*.

[61] Bamford S, Dawson E, Forbes S (2004). The COSMIC (Catalogue of Somatic Mutations in Cancer) database and website. *British Journal of Cancer*.

[62] Lehman TA, Modali R, Boukamp P (1993). p53 Mutations in human immortalized epithelial cell lines. *Carcinogenesis*.

[63] Bartek J, Iggo R, Gannon J, Lane DP (1990). Genetic and immunochemical analysis of mutant p53 in human breast cancer cell lines. *Oncogene*.

[64] Hoe SL, Sam CK (2006). Mutational analysis of p53 and RB2/p130 genes in Malaysian nasopharyngeal carcinoma samples: a preliminary report. *The Malaysian Journal of Pathology*.

[65] Kato S, Han SY, Liu W (2003). Understanding the function-structure and function-mutation relationships of p53 tumor suppressor protein by high-resolution missense mutation analysis. *Proceedings of the National Academy of Sciences of the United States of America*.

[66] Chi SG, DeVere RW, Meyers FJ, Siders DB, Lee F, Gumerlock PH (1994). p53 in prostate cancer: frequent expressed transition mutations. *Journal of the National Cancer Institute*.

[67] Van Bokhoven A, Varella-Garcia M, Korch C (2003). Molecular characterization of human prostate carcinoma cell lines. *Prostate*.

[68] Sayan BS, Sayan AE, Knight RA, Melino G, Cohen GM (2006). p53 is cleaved by caspases generating fragments localizing to mitochondria. *Journal of Biological Chemistry*.

[69] Speidel D, Helmbold H, Deppert W (2006). Dissection of transcriptional and non-transcriptional p53 activities in the response to genotoxic stress. *Oncogene*.

[70] Collavin L, Lunardi A, Del Sal G (2010). P53-family proteins and their regulators: hubs and spokes in tumor suppression. *Cell Death and Differentiation*.

[71] Bode AM, Dong Z (2004). Post-translational modification of p53 in tumorigenesis. *Nature Reviews Cancer*.

[72] Downward J (2004). PI 3-kinase, Akt and cell survival. *Seminars in Cell and Developmental Biology*.

[73] Fu Y, Zheng S, An N (2011). *β*-Catenin as a potential key target for tumor suppression. *International Journal of Cancer*.

